# Organizational Position and Structural Empowerment in Chinese Community Nursing: An Interpretive Case Study

**DOI:** 10.1155/jonm/3611018

**Published:** 2025-08-06

**Authors:** Bo Li, Juan Chen, Natasha Howard

**Affiliations:** ^1^Department of Applied Social Sciences, The Hong Kong Polytechnic University, Hong Kong, China; ^2^Mental Health Research Center, The Hong Kong Polytechnic University, Hong Kong, China; ^3^Saw Swee Hock School of Public Health, National University of Singapore and National University Health System, Singapore; ^4^Department of Global Health & Development, London School of Hygiene & Tropical Medicine, London, UK

**Keywords:** community nursing, nurse empowerment, nursing management, organizational theory, professional relationships, workplace equity

## Abstract

Nurse empowerment is widely recognized for advancing professional development and job satisfaction, yet its structural dimensions remain underexplored in China's community nursing sector. Guided by Kanter's structural empowerment theory, this interpretive case study examines how formal hierarchies and informal organizational dynamics converge to shape community nurses' access to power in nonward settings. Drawing on 24 semistructured remote interviews and unstructured observations in Shenzhen, four interrelated themes were identified: (1) dual-function caregiving roles, (2) entitlement to formal power, (3) variation in informal roles, and (4) inequities in informal power. Each theme explicitly connects to core components of structural empowerment, access to supply, information, support, and opportunities, illustrating how organizational structures and interpersonal relationships jointly shape nurses' empowerment experiences. Findings reveal tensions between formal structures intended to promote empowerment and informal dynamics that sustain power asymmetries. By unpacking these layered power relations, the study offers critical insight into structural empowerment in decentralized nursing contexts and calls for both policy and managerial reforms to foster inclusive, ethically grounded organizational cultures.

## 1. Introduction

Empowerment is a widely cited concept, often associated with human development and self-determination. Originating in social movements aimed at challenging marginalization, it is valued for its transformative potential in reshaping power relations and promoting equity [1].

In nursing research, empowerment has emerged as a key factor in promoting professional development and improving job satisfaction. Its relevance is underscored by three persistent challenges. First, many nursing environments remain inefficient and demoralizing, placing nurses in ethically and emotionally fraught caregiving roles [2, 3]. Second, longstanding power hierarchies between physicians and nurses contribute to professional mistreatment and a sustained sense of inferiority among nursing staff [4]. Third, organizational restructuring driven by technological change has often marginalized nurses' expertise, intensifying struggles for autonomy, recognition, and equitable treatment [5].

The need for nurse empowerment is particularly pressing in countries such as China, where powerlessness continues to constrain nursing practice [6]. The profession is frequently stigmatized as low-status, marked by high workloads, inadequate compensation, and substandard working conditions [4]. Societal preferences for physicians over nurses further compound these issues, eroding the caregiving values central to nursing [7]. In addition, tense doctor–patient dynamics heighten workplace stress, with nurses, such as physicians, often subjected to verbal or physical violence [6].

In Western contexts, empowerment initiatives prioritize professional autonomy and collective bargaining, supported by regulatory frameworks and strong professional unions [8]. For instance, Scandinavian countries such as Finland have implemented nurse-led care models that enhance empowerment by expanding nurses' decision-making authority [9]. Similarly, Magnet hospitals in the United States promote empowerment through participatory governance structures, underscoring the role of supportive leadership and organizational culture [2]. These systemic approaches stand in stark contrast to the hierarchical, resource-limited environments that characterize much of Chinese nursing.

Empowerment remains a pivotal theme in Chinese nursing research, especially within hospital settings. Studies consistently demonstrate its benefits: Ning et al. [10] linked empowerment to improved care quality, Cai and Zhou [11] associated it with higher job satisfaction and reduced turnover, and Guo et al. [12] highlighted its role in mitigating stress and burnout. Lv et al. [13] explored psychological empowerment in the context of fostering innovation, in a way that aligns with Spreitzer's [14] conceptualization of it as a motivational construct comprising meaning, competence, self-determination, and impact.

Hospital nurses work within hierarchical, protocol-driven environments that provide structured resources, clear roles, and specialized teams [12, 13]. Conversely, community nurses operate in decentralized settings with greater autonomy, managing diverse responsibilities across primary care and public health. While such autonomy can enhance professional empowerment by enabling greater decision-making and flexibility [15], it is often tempered by limited institutional support and resource constraints [16]. These contrasting contexts shape distinct experiences and expressions of empowerment in community versus hospital nursing.

Despite its recognized importance, empowerment within China's nursing system remains insufficiently examined. Existing research reveals three critical gaps. First, most studies lack a coherent theoretical foundation; only a few (e.g., [10]) incorporate frameworks such as critical social theory or organizational theory, limiting the development of targeted empowerment strategies. Second, the predominance of quantitative methodologies e.g., [11, 12] constrains a deeper understanding of empowerment's complex, contextualized dynamics, nuances better captured through research that includes qualitative inquiry elements [4]. Third, research on community nurses, who play a pivotal role at the nexus of individual care and public health [17, 18], remains sparse. Given their distinct practice environments compared to hospital settings, community nurses require empowerment approaches tailored to their unique challenges.

Community nursing fundamentally diverges from hospital-based practice in scope, structure, and contextual demands [7]. While hospital nursing focuses on acute, episodic care within regulated environments, community nursing entails broader, longitudinal responsibilities that demand adaptability and negotiation amid resource constraints [17]. In countries such as Canada, community nurse empowerment is fostered through interdisciplinary collaboration and publicly funded healthcare systems, which mitigate workload burdens and enable more equitable resource allocation [19]. These systemic enablers highlight stark contrasts between Western models, characterized by collaborative, resource-rich environments, and the hierarchical, resource-scarce conditions prevailing in Chinese community nursing [18].

This qualitative study addresses these gaps by applying Kanter's [20] structural empowerment theory, which emphasizes how access to organizational power resources shapes individuals' empowerment experiences. Guided by this framework, we investigate Chinese community nurses' perceptions of empowerment within their work environments. Our findings contribute novel theoretical insights into empowerment dynamics in nonward nursing contexts and provide empirical evidence to inform practices aimed at fostering equitable empowerment in community nursing.

## 2. Community Nursing in China

Community nursing in China was formally introduced in 1997 through the Notice on Strengthening Nursing Management [21] and is primarily delivered through community health service centers and stations, which vary in size and service scope [7, 17, 18]. Positioned as a key mechanism in healthcare system integration, community nursing supports a transition from a doctor-centric, treatment-oriented model to a more preventive, population-based approach [21]. By cultivating sustained relationships with local populations, community nurses have become increasingly central to public-facing care.

Under national policy mandates, community nurses are tasked with a broad set of responsibilities, including clinical care, disease prevention, rehabilitation, palliative services, health education, and family planning [17, 18]. These responsibilities reflect the global definition of community nursing as “a synthesis of nursing practice and public health practice applied to promoting and preserving the health of populations” [22].

Despite more than 2 decades of policy support, China has yet to establish a fully robust community nursing system [7]. Public trust in community nursing remains limited; patients frequently question the competence of community-based providers and may avoid their services altogether [23]. These trust deficits, combined with structural limitations, have impeded the professional empowerment of community nurses.

China's tiered healthcare structure further entrenches hierarchical disparities, positioning community nurses at the base of the system [4]. Unlike hospital-based nurses who derive authority from clinical specialization [24], community nurses often lack recognized expertise due to the generalist scope of their work [4, 18]. Given that professional authority is closely tied to specialization [25], this generalist positioning contributes to their systemic marginalization [17].

Strengthening the empowerment of community nurses is essential for advancing population health goals [18]. Since the launch of the Healthy China Initiative in 2012, the role of community nurses in preventive care and chronic disease management has been increasingly emphasized [4]. The latest National Nursing Development Pla*n* [26] further underscores the need to reduce intraprofessional disparities and enhance the infrastructure of community-based care. However, the actual extent of empowerment experienced by community nurses remains insufficiently understood, warranting an in-depth examination of their everyday work environments and organizational positioning.

## 3. Theoretical Framework

This study adopts an organizational lens, using Kanter's [20] structural empowerment theory to examine power dynamics within community healthcare settings. Drawing on fieldwork in a major American corporation, Kanter [20] defines power as “the ability to mobilize human and material resources to get things done.” Moving beyond traditional conceptions of power as control, dominance, or coercion, she reframes it as a productive organizational force characterized by efficacy, capacity, and fulfillment. This redefinition supports her argument for fostering empowerment rather than containing power within workplace structures.

Kanter [20] contends that power derives primarily from an individual's organizational role rather than personal characteristics. She distinguishes between formal and informal positions within the organizational structure. Formal positions involve job-related functions, including decision-making authority, recognition for performance, and alignment with institutional goals. Informal positions arise from interpersonal relationships within the hierarchy, such as support and delegation from superiors, collaboration and resource-sharing with peers, and assistance from subordinates, who may alleviate workload and serve as proxies for leadership.

Access to organizational resources, a core component of Kanter's theory, is shaped by both formal and informal roles. These resources include the following: the ability to obtain materials, funding, recognition, or status necessary to meet professional and institutional goals (supply); access to critical knowledge and communication channels (information); endorsement from key stakeholders that fosters autonomy, innovation, and effectiveness (support); and opportunities for career advancement and skill development (opportunity). Individuals occupying higher-status formal or informal roles typically enjoy greater access to these resources, reinforcing their power, whereas those in lower-tier positions face a heightened risk of disempowerment.

This study applies Kanter's theory to examine the organizational positioning of community nurses, guided by two research questions: (1) What formal and informal roles do community nurses occupy within their organizations? (2) How do these roles influence their access to organizational power resources and their experiences of workplace empowerment?

## 4. Methods

### 4.1. Design

To capture the complexities of social power [4], we employed an interpretive case study approach. This design, grounded in established theoretical frameworks, enables nuanced and contextually informed insights aligned with the study's what–how research questions [27].

The study was conducted in Shenzhen, a key site for China's community health reforms and policy innovation [4, 17, 18]. Over the past 3 decades, Shenzhen has developed a comprehensive community healthcare system comprising community hospitals, health service centers, and stations. Community hospitals, launched in late 2022 under the Capacity Expansion and Quality Improvement Initiative [28], provide emergency and inpatient care. Meanwhile, health service centers and stations deliver primary care and general practice services. By 2022, Shenzhen employed nearly 5000 nurses across over 750 community health agencies [29]. This extensive network offers an optimal setting for fieldwork and rich data collection [27].

We defined the “case” as Shenzhen's community nursing service a bounded context within the healthcare system where nurses' professional roles, resource access, and relational dynamics are shaped by the city's healthcare infrastructure and reform efforts. Focusing on this unit of analysis allows examination of the interplay between organizational power, formal and informal roles, and resource distribution. Shenzhen's mature healthcare framework thus provides locally grounded insights with broader relevance to urban healthcare systems [18].

### 4.2. Participants

Participants were purposively recruited based on eligibility criteria including a minimum of 1 year's full-time community nursing experience, representative of the majority of Shenzhen's community nursing workforce [7, 17]. Active employment as a community nurse in Shenzhen was also required to ensure direct relevance to the study focus. To mitigate potential bias arising from administrative hierarchies, managerial head nurses were excluded, prioritizing frontline nurses engaged in direct community health service delivery and grounding the analysis in their lived experiences.

Recruitment was facilitated by BL through a WeChat group consisting of approximately 40 community nurses who had participated in prior research [4, 7, 17, 18]. This group was initially formed in 2021 when BL invited nurses to share their WeChat contact details to enable efficient communication amid COVID-19 restrictions on face-to-face interactions. The network subsequently expanded through peer referral during follow-up studies, fostering an engaged cohort familiar with the research context.

A recruitment notice detailing study objectives, procedures, incentives, and confidentiality was disseminated. Within two weeks in January 2023, 26 nurses expressed interest; two were excluded due to ineligibility (e.g., not currently practicing in Shenzhen). Between March and December 2023, 24 nurses (Table 1) completed semistructured, open-ended interviews via WeChat video calls [30]. Participants received an RMB 100 honorarium via WeChat as a token of appreciation for their time.

### 4.3. Data Collection and Analysis

The semistructured interview guide (Table 2) was developed with reference to Kanter's theory. It was designed to address the study's two research questions by examining the formal and informal positions nurses held and how these positions shaped their access to organizational power resources. While theoretically grounded, the guide retained flexibility to pursue emergent lines of inquiry. All interviews were conducted in Mandarin by BL, lasted 70–90 min, and were audio-recorded with participants' consent.

During interviews, we documented both verbal responses and nonverbal cues [32]. Expressions of frustration, anger, fear, discomfort, happiness, and satisfaction frequently emerged when participants discussed interpersonal dynamics in informal roles. These nonverbal signals guided follow-up questions and deepened insight into participants' experiences. Incorporating both verbal and nonverbal data from video recordings enriched the dataset, facilitating semantic and latent coding.

Guided by Kanter's theory, we employed theory-informed (deductive) thematic analysis [33]. This approach, well-suited for studies grounded in established theory, enabled us to apply predefined themes while remaining receptive to emergent insights, allowing each to inform the other. Kanter's framework provided a lens to identify themes concerning formal and informal roles and their influence on access to organizational power.

We began by autotranscribing video files and thoroughly reviewing transcripts to grasp participants' narratives. We then identified central concerns, typical cases, and outliers. Saturation was monitored throughout coding by continuously comparing new data with existing codes and themes [34]. By the 19th interview, no new codes or significant thematic variations emerged, indicating comprehensive coverage of participants' experiences. This was confirmed in the final coding round, where additional data reinforced rather than expanded the established themes.

We employed a theory-driven coding approach, guided by Kanter's key concepts. BL conducted thematic coding using ATLAS.ti, applying codes deductively based on predefined theoretical constructs. Data that did not fit these themes were examined for emerging patterns. Regular team discussions ensured coding consistency and minimized potential biases.

As we organized codes to identify shared meanings rooted in Kanter's concepts of formal and informal roles and resource access, we remained receptive to insights that expanded or challenged these frameworks. This iterative process enabled a nuanced synthesis of interpretations aligned with the themes. In the final stage, these interpretations were refined during manuscript drafting and revision to ensure a coherent and rigorous presentation, consistent with O'Brien et al.'s [35] SRQR guidelines.

### 4.4. Reflexivity

We recognize that our social positions, including BL's identity as a male researcher and our international institutional affiliations, influenced the research process. Reflexive dialog was maintained throughout the study to acknowledge and mitigate these influences, thereby enhancing transparency and interpretive rigor.

Informed by prior research on power dynamics in Chinese community nursing, we were aware of the structural marginalization that community nurses often face within hierarchical healthcare systems. While we acknowledged this prior knowledge, we made a concerted effort to bracket our assumptions and remain open to participants' narratives. This reflexive stance supported an analysis grounded in participants' lived experiences, rather than being shaped by preexisting theoretical or epistemological commitments.

### 4.5. Ethics

Ethical approval was obtained from the Institutional Review Board of The Hong Kong Polytechnic University prior to data collection. Oral informed consent was secured from all participants. Confidentiality was maintained through adherence to institutional data security protocols governing the storage, transfer, and disposal of video recordings and transcripts. All personal identifiers were removed, and anonymized codes were used in reporting.

## 5. Findings

**Figure 1:**
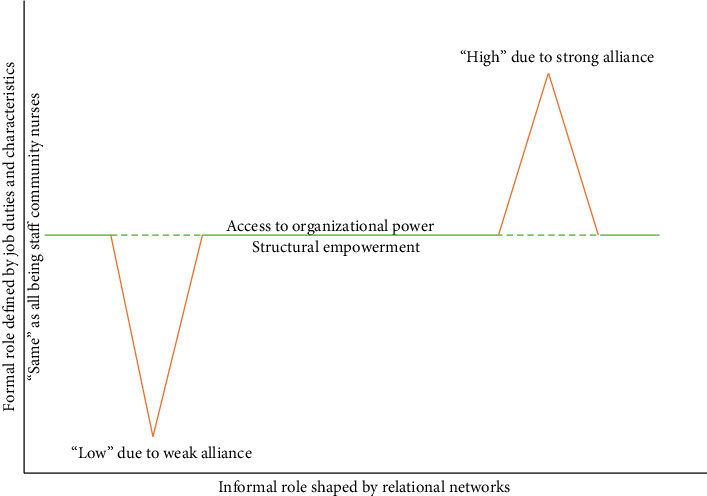
Power dynamics of community nurses within dual-role structures. Note: This figure synthesizes key findings by illustrating the formal and informal roles held by community nurses, the factors shaping these roles, and their implications for empowerment. Green lines denote “equal” formal access to organizational power; however, the dotted lines signify that such access is mediated by differences in informal roles shaped by interpersonal ties. These variations are depicted by orange lines, indicating uneven empowerment, nurses with stronger alliances gain greater access to power, while those with weaker networks face constraints. The figure also highlights how informal roles influence the degree of power nurses derive from formal positions. In community health service centers, characterized by flat hierarchies and limited staffing [4, 18], the interplay between formal and informal roles was less pronounced than in-hospital settings. Still, within Chinese organizational culture, formal and informal roles remain deeply interlinked: higher formal status often translates into stronger informal influence, and vice versa, through mechanisms of authority and social support.

Our analysis generated four interrelated themes: (1) dual-function caregiving roles, (2) entitlement to formal power, (3) variation in informal roles, and (4) inequities in informal power. Together, these themes offer a conceptual framework for understanding community nurses' power dynamics within a system of nested, dual-role expectations (Figure 1).

### 5.1. Dual-Function Caregiving Roles

Community nurses formally occupy frontline caregiving roles that encompass dual care functions, clinical and public health, that define their professional activities and roles.

The first dimension involves direct patient interactions at community health service centers, positioning nurses as the first point of contact in care delivery. IW5 referred to themselves as “stormtroopers” on the front lines, while IW1 emphasized, “This is a healthcare organization where patient care is my foremost priority.” IW4 similarly noted, “We're always at the forefront, dealing with the most immediate health concerns.”

The second dimension includes broader responsibilities beyond clinical care. As IW6 explained: “My routine involves vaccinations, injections, and assisting physicians with dressing changes. In addition, I engage with the community through outreach programs promoting health literacy, smoking cessation, and food safety. These nonclinical services are equally critical to my role.” IW7 echoed this duality: “It's a unique position because you're expected to provide clinical care, but just as importantly, you're educating the community on preventive health and guiding them on lifestyle changes.”

This integration of clinical and public health tasks was consistently reflected in participants' accounts, indicating strong alignment with institutional expectations for comprehensive community-based care.

However, the balance between these functions varied across individuals, often shaped by gendered assumptions. IW10 shared: “In theory, my responsibilities include both clinical and nonclinical services, but in practice, I rarely perform clinical tasks beyond specific cases, such as dressing changes for male patients. Female nurses are often seen as more suited for general patient care. As a result, I primarily handle nonclinical tasks like health education, which are perceived as requiring more physical strength.”

This distribution was not merely pragmatic but embedded in gendered norms regarding perceived suitability for care work. As IW11 explained: “Some tasks, like comforting elderly patients or doing health assessments, are believed to be better handled by female nurses. Male nurses are often assumed to be more assertive or strong, so we get asked to lead community talks or manage logistics for outreach events.”

These accounts suggest that male nurses are often directed toward nonclinical responsibilities that are more public-facing or managerial in nature, reinforcing gendered divisions of labor and differential access to informal power. While female nurses tend to be more involved in direct patient care, male nurses are frequently positioned, intentionally or not, as organizers or health educators, shaping perceptions of authority and influencing distinct professional trajectories.

This theme underscores the structural duality of community nursing roles while illuminating the gendered nuances that shape task allocation and professional identity in practice.

### 5.2. Entitlement to Formal Power

Community nurses' formal roles position them as key agents in achieving organizational goals, granting access to structural power consistent with Kanter's framework.

First, their duties are central to improving health outcomes and community well-being. As IW18 explained, “Our main objectives are to reduce preventable diseases through immunization and health education, directly aligned with the organization's mission.” IW12 reinforced this, noting, “Achieving targets like hypertension management or vaccination coverage shapes how the community views our quality of care.” Such contributions are formally recognized through organizational rewards, both material and symbolic. IW7 recounted, “During COVID-19, my salary increased, and I was named an “excellent community-health worker,” which motivated me and enhanced my reputation.” IW9 similarly reflected, “Financial bonuses after immunization campaigns boosted morale and affirmed our value to the organization.”

Second, community nurses balance clinical excellence with advanced nonclinical responsibilities. Their expertise is considered “an invaluable collective asset” (IW20), prompting investment in targeted training. IW16 shared, “I regularly attend specialized sessions on specimen collection and palliative care, which enhance my skills.” IW10 added, “Workshops and certification programs keep us updated on best practices and innovations.”

Third, their dual functions require interdisciplinary collaboration. Nurses work closely with “general practitioners, dispensers, acupuncturists, and lab technicians” (IW19), while also serving as “community health cadres for nonclinical duties” (IW4). IW1 highlighted, “Unlike hospital nurses, we operate across departments and handle diverse tasks, from immunizations to blood draws and pharmacy work, broadening our skills and professional networks.” These interactions provide vital informational power and expand their influence.

Finally, effective service delivery depends on professional supervision. Participants noted that “training and textbooks alone are insufficient” (IW22), emphasizing the importance of mentorship from “family-doctor teams led by attending physicians or general practitioners” (IW24). IW3 explained, “Supervision is crucial for newcomers to reduce errors and build confidence, while experienced nurses rely less on oversight.” IW15 affirmed, “Veteran nurses like me handle challenges independently, needing minimal supervision.”

Despite their formal empowerment, gender and experience influence access to organizational power. Limited clinical involvement can isolate male nurses from cross-departmental exchanges, restricting informational power. IW13 remarked, “My focus on public health tasks like paperwork limits my interaction with clinical colleagues, sometimes causing detachment.” IW14 observed, “Focusing on data analysis and population-level interventions means missing out on dynamic clinical teamwork, a professional trade-off.”

These findings reveal how formal roles confer organizational power while exposing subtle variations shaped by gender, experience, and task allocation within community nursing.

### 5.3. Variation in Informal Roles

Kanter highlights that informal organizational positions arise from interpersonal relationships and colleague support, shaping access to power resources. Applying Kanter's concept of alliances, networks linking nurses with superiors and peers, our findings show that all participants occupied informal roles shaped by these relationships. We identified two alliance types distinguished by the level of support they offered, which influenced nurses' informal power.

The first type, strong alliances, feature influential leaders who wield power both upward, securing recognition and rewards from higher management, and downward by delegating authority and resources to empower members. IW17 shared, “I've built a close relationship with our head nurse, fostering trust. She assigns me key tasks and allocates resources, and beyond work, we are friends who share insider organizational information.” IW3 echoed this: “Influential colleagues open doors for resources and career growth. Their support and trust make our alliance strong.”

Participants described several strategies they employed to cultivate and sustain strong alliances. One key approach was proactive engagement, such as volunteering for additional responsibilities, which signaled commitment and built trust with superiors. As IW3 noted, “I always offer to take on more complex tasks when the team is overwhelmed. Leaders notice that, and it's helped me gain their support.” Informal socialization beyond work hours also emerged as a vital practice for fostering rapport: “Joining after-work gatherings or even sharing lunch together makes a big difference,” explained IW17. “It helps build personal connections.” Consistency and reliability were likewise viewed as foundational qualities. IW5 reflected, “You need to prove you're dependable. Once your colleagues see that, they'll start including you in important discussions or decisions.”

Strong alliances also involve professional diversity, essential in community healthcare's cross-departmental context. IW5 noted, “Building relationships across departments expands your network, which is invaluable when you need assistance. I maintain ties with colleagues in other specialties, improving communication and access to their resources.”

In contrast, weak alliances stem from limited connections with superiors and professional homogeneity. Weak ties to leadership reduce access to top–down support critical for power. IW8 explained, “I have close colleagues but none in managerial roles. We support each other, but our capacity is limited. Managers prioritize those they have personal ties with, especially for training opportunities. If one of us were a manager, things would likely be different.”

Participants embedded in weaker alliances frequently described experiences of professional stagnation, limited access to development opportunities, and emotional strain. As IW12 reflected, “It's disheartening when you know you're capable, but you're always the last to be considered for workshops or special projects. You begin to question your value here.” The absence of supportive leadership also compromised psychological safety, discouraging staff from raising concerns or proposing innovations. IW24 shared, “When you're not close to anyone in charge, it's risky to speak up… so most of the time, I just stay silent.” Feelings of exclusion from informal networks further intensified emotional burdens. IW11 observed, “You see some colleagues going out together after work, exchanging news and opportunities, it's like a circle you're not part of… it can feel very isolating.” These accounts underscore how weak alliances not only constrain access to power resources but also reinforce organizational hierarchies, diminishing morale and potentially impacting staff retention.

All-nurse alliances, while fostering “belonging” (IW8), lack external influence and cross-departmental reach. IW2 lamented, “We get along well but don't hold influential positions, so our relationships don't grant access to resources or ease our tasks” (expressed with a sigh and a shake of her head). Without structural ties to influential actors, these alliances often function more as support networks than sources of power, rich in camaraderie, but limited in organizational impact.

Age and seniority bolster informal power. In Chinese culture, seniority commands respect, enabling older nurses to connect more easily with influential leaders. Their “legitimate power” [4] enhances peer esteem and professional appeal, broadening networks and informal influence. IW16 reflected, “Being older gives me an advantage, people respect my experience, and younger colleagues consult me. This respect helps me connect with senior leaders and grow my network.” Several participants described how accumulated experience functioned as symbolic capital, facilitating navigation through workplace hierarchies and fostering trust. IW20 shared, “When I first started, it was hard to know who to ask for help. Now, after more than a decade here, I've built relationships across departments. People listen to me more, and I can offer guidance to newer staff.” Similarly, IW21 reflected, “Seniority means I've seen different leaders come and go, I've learned how to adapt, who to approach, and when to speak up. That kind of intuition only comes with experience.” These reflections underscore that age and tenure are not merely markers of seniority, but active resources that shape relational dynamics and the accrual of informal power within the organization.

This theme underscores variation in informal roles, revealing how personal attributes, age and experience, and relational dynamics shape nurses' informal power within the organization.

### 5.4. Inequities in Informal Power

While nurses' formal roles ostensibly grant them access to organizational resources, our findings indicate that actual access is highly contingent on informal positions shaped by interpersonal dynamics. Those who cultivate strong alliances often benefit from enhanced entitlement, gaining privileged access to informal power resources such as supplies, insider information, professional support, and career advancement. These nurses are able to navigate and leverage less visible organizational processes to maintain influence and accumulate informal power over time. As IW1 critically observed, “One of my colleagues has a close relationship with the head of our center, which gives her early access to information and insights that the rest of us don't get. While this benefits her, it clearly creates an imbalance, and others perceive it as unfair.” Similarly, IW13 remarked, “Having a direct line to someone in a decision-making role changes everything, suddenly, tasks get easier, and opportunities come your way without even asking.”

These alliances also shaped nurses' professional identity and confidence. Those embedded in strong networks often reported greater job satisfaction and optimism about their career trajectories. As IW10 reflected, “When your supervisor trusts you and involves you in decision-making, you feel valued, it motivates you to grow.” IW7 similarly shared, “Because my mentor has a good relationship with the head nurse, I was recommended for additional training. That kind of support makes a real difference.”

Conversely, nurses with weaker interpersonal connections reported more limited access to informal resources and reduced opportunities for professional growth. IW8 shared, “It's rare to quickly build trust with influential figures, and even when you do, the existing power dynamics remain hard to overcome.” These individuals often find themselves positioned lower in the informal hierarchy, constrained by limited influence and compelled to accept disparities as a fixed part of the organizational landscape. As IW4 put it, “Even though we all do the same job, there's no real equality when it comes to access to resources. The ones with close relationships with the management get priority, especially when resources are scarce. As a staff nurse without any influence, I have no choice but to accept this unfair treatment.” IW9 echoed this sentiment: “It's frustrating because no matter how hard you work, it seems like the ones with personal connections always end up ahead. Effort alone doesn't count as much as who you know.”

For some nurses, the absence of supportive alliances led to demotivation and a sense of career stagnation. IW1 shared, “When you're not part of the inner circle, it's easy to feel invisible. I've thought about transferring because I don't see a future here without connections.” Such accounts underscore how limited access to informal networks not only hinders professional development but also diminishes morale and long-term organizational commitment.

Notably, perspectives on informal power inequities diverged by generational position. Younger nurses frequently described these dynamics as oppressive, expressing a sense of marginalization within the hierarchical structure. In contrast, older nurses, who often benefit from seniority and greater access to informal influence, did not report similar concerns. Their proximity to power appeared to temper critiques of systemic inequality. As IW21 commented, “I don't see any disparities in access to resources. As far as I'm concerned, they're equally available to everyone. It's not about who can or can't access them; we're all nurses and should be treated as equals.”

Beyond perceived fairness, age and experience also shaped nurses' career trajectories. Several younger participants expressed frustration with the slow pace of advancement despite consistent performance. IW1 noted, “Sometimes I feel stuck. Even though I've worked hard, the older nurses always get considered first for new roles or training. It's like experience outweighs potential.” In contrast, older nurses often described a smoother path, facilitated by accumulated trust and organizational familiarity. As IW21 explained, “After working here for years, I know who to talk to and how things work. That's helped me move forward without needing to push too hard.” These accounts suggest that while experience provides valuable institutional knowledge, it may also reinforce age-related advantages that hinder younger nurses' upward mobility.

These findings illuminate how informal power is unevenly distributed across the organization, shaped by individual access to relational capital and reinforced by age and seniority. Despite formal equality in role definitions, significant disparities persist in how resources, recognition, and advancement opportunities are distributed, raising critical questions about equity and fairness in everyday organizational life.

## 6. Discussion

This study employed Kanter's [20] structural empowerment theory to explore how community nurses in China experience empowerment within their organizational environments. Four interrelated themes emerged: (1) dual-function caregiving roles, (2) entitlement to formal power, (3) variation in informal roles, and (4) inequities in informal power. Together, these themes reveal that access to structural empowerment, comprising supply, information, support, and opportunity, is mediated not only by formal systems but also by informal dynamics, including interpersonal alliances, seniority, and relational capital. While some nurses leverage formal positions and strong networks to enhance their influence, others are constrained by hierarchical norms and limited access to informal structures. By situating these findings within Kanter's framework, the analysis highlights how structural and relational forces intersect to produce uneven experiences of professional development, resource distribution, and workplace inclusion.

### 6.1. Key Findings

This study contributes original insights by examining the nuanced informal roles of community nurses within organizational settings. Our findings reveal a diverse spectrum of interpersonal relationships that intricately shape nurses' informal positions, echoing Tripathy's [36] observations on relational complexity. Drawing on Kanter's [20] concept of alliances, this study elucidates the underlying psychological drivers of these interpersonal practices, rooted in fundamental human needs for belonging, attachment, and affiliation [37]. This theoretical framing advances understanding of how informal relational dynamics influence nurses' organizational roles and access to power. Although nursing relationships have been extensively explored [38], our analysis introduces a dichotomous perspective on nurse allyship, distinguishing between strong and weak alliances, thereby offering a novel lens to interrogate these interpersonal processes.

This study underscores the complexity of informal interpersonal relationships within organizational contexts, particularly in the Chinese cultural setting. These dynamics arise from both implicit cultural norms and explicit organizational hierarchies, shaping access to resources through the cultivation or limitation of social capital, trust, and collaboration [39]. For instance, Confucian values emphasizing hierarchy and the concept of “guanxi” [40] play a crucial role in forming alliances, which in turn influence resource allocation and decision-making processes. Recognizing and addressing these culturally embedded dynamics is vital for effective human resource management in community health services.

The distinction between formal and informal roles is central to this study's analysis of structural empowerment in community nursing. Introducing the concept of entitlement clarifies how these roles shape nurses' access to power. Formal roles, defined by organizational structures and job responsibilities, provide nurses with inherent access to essential resources. This form of empowerment challenges critical theory's traditional view of empowerment as a solely top–down process [41], instead positioning it as an intrinsic element of professional practice and identity.

While diverging from critical theory, the findings align with Kuokkanen and Leino-Kilpi's [1] assertion that nursing is fundamentally rooted in human relationships, consistent with the philosophy of care [42]. This study suggests that human agency shapes the extent, rather than the form, of empowerment. Hierarchical empowerment manifests in two key ways: superiors control the scope of empowerment by granting resource access through alliances, while peers contribute through networking and social capital. These findings broaden the traditionally top–down focus in empowerment literature [41], providing a foundation for further research on the dynamics of organizational empowerment.

To critically engage with alternative empowerment frameworks, this study underscores the importance of shared governance models, which emphasize collective decision-making and egalitarian collaboration within nursing teams [43]. Unlike Kanter's structural approach, shared governance prioritizes participatory empowerment, highlighting shared accountability in fostering equitable practices. Integrating these perspectives offers a promising avenue for future research to explore how structural and participatory empowerment intersect to shape nurses' experiences and organizational outcomes. Furthermore, intersectionality theories can deepen understanding of how overlapping social identities, such as gender, age, and professional status, either exacerbate or mitigate empowerment disparities [44].

The study also reveals nuanced experiences of empowerment shaped by age and gender. Older nurses often benefit from age-related advantages that strengthen their informal roles, thereby deepening inequities in empowerment practices. Conversely, male nurses encounter distinct challenges, as their roles typically focus on primary and general care, areas traditionally viewed as less masculine compared to emergency or intensive care [4, 24]. Coupled with their minority status within the nursing workforce [7], these factors restrict equitable empowerment for male nurses. Further comparative research examining male nurses in both community and hospital settings is warranted to better understand and address these disparities.

This study's focus on informal power dynamics in China highlights the profound influence of cultural norms on professional relationships. In Chinese nursing, Confucian values prioritize seniority, hierarchy, and respect for authority [4], granting older nurses privileged access to resources and decision-making networks, while younger nurses often face informal barriers. This contrasts with more individualistic contexts where informal power tends to stem from personal achievement, expertise, or meritocratic networks rather than age or tenure [45]. For instance, American nurses often form informal alliances based on shared specialties or leadership potential [46], rather than hierarchical status. Similarly, Scandinavian healthcare settings emphasize flat organizational structures and egalitarian values [47], promoting a more equitable distribution of informal power across age groups. These cross-cultural differences underscore that informal power is socially constructed and deeply embedded in local values and organizational norms. Comparative research is essential to better understand how culture shapes informal power and to develop culturally sensitive strategies for fostering equitable empowerment in nursing worldwide.

### 6.2. Policy and Institutional Implications

To unlock the full potential of community nursing in advancing population health, it is essential to address structural inequities in empowerment. This study offers targeted policy and organizational strategies to support more equitable empowerment within China's community nursing system.

Community nursing remains constrained by systemic inefficiencies and stigma associated with its marginal status [7, 21]. Participants reported fragmented service delivery, resource shortages, and professional undervaluation, all of which undermine nurses' ability to perform their roles effectively. Moreover, organizational dynamics often privilege senior staff or those with strong informal alliances, marginalizing junior or socially isolated nurses. Without policy safeguards, these informal hierarchies perpetuate unequal access to advancement and resources. To address this, policies should formalize community nursing roles through standardized job descriptions, ensure fair resource distribution, and invest in structured mentorship programs for early-career nurses. Funding models and performance metrics should also incentivize interdisciplinary collaboration and team-based care. Including community nurses in policy development processes can elevate their professional visibility and ensure reforms reflect frontline realities.

Yet policy alone is insufficient to redress organizational inequities. Structural and cultural changes are also needed, with leadership playing a central role. Effective leaders must adopt inclusive, ethical practices that reject autocratic and patriarchal norms [45]. Participants emphasized the importance of transparent decision-making and equitable resource allocation in fostering agency and ownership among staff. While informal alliances are critical, they cannot be imposed, trust and collaboration must evolve organically. As one nurse (IW5) observed, “Trust takes time and cannot be forced, it grows from shared goals and mutual respect.”

To reduce disparities in informal power, organizations should implement transparent criteria for resource allocation and role assignment, minimizing favoritism based on seniority or social ties. Inclusive mentorship initiatives are crucial for bridging experience gaps and fostering belonging [48]. Given the gendered and age-related dynamics in community nursing, leadership development programs should account for the specific needs of younger or male nurses, who may face greater barriers to influence within informal networks. Regular audits of empowerment practices can help identify and dismantle persistent structural barriers.

Rather than mandating alliance formation, leadership should cultivate collaborative environments through team-building and peer mentorship. Clear communication channels, feedback loops, and conflict resolution mechanisms are essential to building an equitable culture. Leaders must also bridge the disconnect between policy directives and on-the-ground realities by incorporating frontline perspectives into decision-making. Doing so ensures that empowerment strategies are both relevant and sustainable.

Finally, collaboration across policymakers, educators, and managers is critical. Policymakers should establish governance frameworks that promote equitable access to resources and fund leadership development in community settings. Educators can prepare nurses to navigate hierarchical systems by embedding empowerment theories and skills into training curricula. Managers must operationalize these strategies by fostering inclusive workplaces and regularly evaluating empowerment initiatives. Coordinated efforts across these sectors are key to embedding lasting, equity-driven reforms in community nursing.

### 6.3. Limitations

This study acknowledges five key limitations. First, potential gender bias may have influenced the findings. Male community nurses, already a minority in China, were underrepresented due to recruitment constraints. This limited our ability to fully explore how gender shapes informal roles and empowerment. Given established gender-based differences in interpersonal dynamics [49], the underrepresentation of male voices restricted a more nuanced gender analysis. In addition, our purposive sampling strategy, recruiting via a WeChat group of nurses previously involved in related research, may have introduced selection bias. Participants were likely more familiar with the research team and potentially more attuned to empowerment discourses, which could have influenced their responses. While this approach was pragmatic given logistical and access constraints, it limited the diversity of professional roles and career trajectories represented. Future studies should consider broader recruitment strategies, such as stratified or random sampling across institutions and career stages, to improve representativeness and mitigate bias.

Second, while online interviews facilitated geographically diverse participation, the lack of face-to-face interaction constrained our ability to observe nonverbal cues and implicit relational dynamics. This limitation may have reduced insight into how informal power is enacted in everyday interactions. Future research should consider hybrid designs incorporating in-person interviews or ethnographic observation to better capture these subtle dynamics.

Third, the absence of detailed data on participants' educational backgrounds limited contextual analysis. Educational attainment can shape nurses' perspectives, decision-making capacity, and access to empowerment [50]. Including this variable in future studies would enhance understanding of how education intersects with organizational power structures.

Fourth, while Kanter's empowerment theory provided a valuable foundation, exclusive reliance on this framework may have narrowed interpretation. For example, younger nurses lacking strong informal alliances were primarily viewed as disempowered; however, alternative frameworks, such as critical resistance theory, might interpret their positions as expressions of agency within hierarchical systems. Broader theoretical integration would enrich future analyses of empowerment in nursing.

Finally, the study's focus on Shenzhen limits transferability. Shenzhen's relatively advanced healthcare infrastructure and reform-oriented context may not reflect conditions in rural or underresourced regions. Consequently, findings should be interpreted with caution beyond this setting. Comparative research across diverse health systems is needed to explore context-specific and generalizable empowerment dynamics.

## 7. Conclusion

Community nursing, described as “the lifeboat to the health services' Titanic” [51], plays a pivotal role in advancing universal health coverage and transforming care delivery. Despite growing global recognition of community nurses' contributions to individual and population health, their roles in China remain structurally marginalized within a hierarchical healthcare system. While their caregiving functions are increasingly described, their broader organizational value and professional status remain underexamined.

Drawing on Kanter's structural empowerment theory, the study examines how organizational position and resource access shape empowerment among Chinese community nurses. It introduces a nested dual-role framework, revealing a disconnect between formal empowerment mechanisms and informal power dynamics that often reinforce inequity. These insights underscore the importance of addressing both structural and relational dimensions of empowerment.

Although grounded in the Chinese context, the findings speak to broader organizational challenges in nursing, particularly around power asymmetries, role legitimacy, and informal influence. By highlighting how interpersonal alliances and informal hierarchies shape empowerment, the study offers transferable insights relevant to diverse healthcare settings.

Empowering nurses is essential not only for enhancing care delivery but also for promoting workforce well-being and organizational resilience. While nursing is often positioned as peripheral to institutional power, this study reveals its embedded influence, both formal and informal, within healthcare systems. However, disproportionate informal power concentrated in selected individuals can undermine equity and collaboration. These findings call for a more nuanced, context-sensitive approach to nurse empowerment, one that balances authority, participation, and accountability within inclusive and equitable organizational cultures.

## Figures and Tables

**Table 1:** Participants.

Code^a,b,c^	Age	Sex^d^	Years of service^e^
IW1	25	Female	3
IW2	29	Female	3
IW3	24	Female	2
IW4	31	Female	5
IW5	25	Female	2
IW6	32	Female	5
IW7	25	Female	1
IW8	26	Female	3
IW9	28	Female	4
IW10	26	Male	2
IW11	23	Male	1
IW12	23	Male	2
IW13	27	Male	3
IW14	25	Male	3
IW15	41	Female	14
IW16	37	Female	9
IW17	42	Female	12
IW18	37	Female	10
IW19	36	Female	8
IW20	40	Female	7
IW21	38	Female	13
IW22	26	Male	2
IW23	25	Male	3
IW24	27	Female	5

^a^Participants were assigned codes sequentially based on their interview order.

^b^Participants were recruited from 11 community health centers in Shenzhen, each serving 10,000–60,000 residents across 0.4–5.2 km^2^ and staffed by 14–40 full-time health professionals.

^c^Educational background was not examined, as most community nurses in Shenzhen hold similar postsecondary vocational diplomas [7, 17]. In this context, work experience is valued more than part-time training and is the primary basis for professional recognition.

^d^Male nurses are a minority in community healthcare in China, reflecting gender norms and sector-specific role expectations and status [7, 31].

^e^Years of experience refer to the total time working within Shenzhen's community healthcare system.

**Table 2:** Interview guide.

Theoretical dimension	Research question addressed	Core interview questions and prompts
Formal and informal roles (framing construct)	What formal and informal roles do community nurses occupy within their organizations?	° How would you describe your current role and responsibilities?
		° What kind of tasks do you typically handle?
		° Are there any tasks you take on that are not officially assigned?
		° How are decisions made in your team, and what is your role in those decisions?

Supply	How do these roles influence their access to organizational power resources and their experiences of workplace empowerment?	° What resources (e.g., time, staffing, and materials) are available to help you do your job?
		° How do you access those resources?
		° Are there any constraints that affect your ability to obtain what you need?
		° Who controls access to these resources?
Information		° How do you stay informed about decisions or changes in your organization?
		° Who shares important information with you, and how?
		° Are there situations where you feel left out of the loop?
Support		° What kind of support do you receive from supervisors, peers, or other professionals?
		° Who do you turn to when facing difficulties at work?
		° Are you comfortable seeking help or advice when needed?
Opportunity		° Have you had chances to grow or advance in your role?
		° Are there opportunities for professional development, training, or promotion?
		° What influences whether you take up those opportunities?

## Data Availability

Participants were assured that raw data would remain confidential and not be shared. Further information about the study is available upon request.

## Supporting Information

The attached file demonstrates our adherence to the Standards for Reporting Qualitative Research (SRQR).
