# Supplementary material for: Organizational Position and Structural Empowerment in Chinese Community Nursing: An Interpretive Case Study
Source: J Nurs Manag. 2025 Aug 6;2025:3611018. doi: 10.1155/jonm/3611018 (PMC12349986; doi:10.1155/jonm/3611018)
Supplement: Supporting Information — Additional supporting information can be found online in the Supporting Information section. [file 3611018.f1.docx]

Table S1. Compliance with the Standards for Reporting Qualitative Research (SRQR) in the study “Organizational Position and Structural Empowerment in Chinese Community Nursing: An Interpretive Case Study”

| SRQR Guidelines | | The study |
| --- | --- | --- |
| No. | Topic |  |
| Title and abstract | | |
| S1 | Title | We designed our Title to concisely reflect the study’s nature, including its context, purpose, and method. |
| S2 | Abstract | The Abstract we provided includes the main aspects of the study, covering the background, objective, methods, findings, and conclusions. |
| Introduction (and background/conceptual framework) | | |
| S3 | Problem formulation | The Introduction section begins with an overview of empowerment in nursing, followed by an outline of the current state of empowerment among nurses in China. It then reviews relevant studies to identify three existing gaps. Additionally, we provide detailed information about community nursing in China to give non-Chinese readers a better understanding of the research background. |
| S4 | Purpose or research question | Based on the synthesis of Kanter’s theory, we formulated two interrelated research questions to guide the study. This theory-driven approach justifies our selection of the interpretive case study as the research method. |
| Methods | | |
| S5 | Qualitative approach and research paradigm | We specified our qualitative approach as an interpretive case study and provided justification for this method. |
| S6 | Researcher characteristics and reflexivity | We underscored how the composition of the team contributed to the study’s development in the Reflexivity section. |
| S7 | Context | We detailed the study setting and rationalized our focus on Shenzhen for the study. |
| S8 | Sample strategy | We delineated our sampling methodology for participant recruitment, elucidating the rationale behind our approach. |
| S9 | Ethical issues pertaining to human subjects | We detailed the measures taken to uphold ethical standards in research involving human subjects. |
| S10 | Data collection methods | We provided a comprehensive description of the data collected, along with a detailed outline of the process and methods employed for data collection. |
| S11 | Data collection instruments and technologies | We elaborated on the interview guide utilized for data collection and highlighted the methodology of conducting interviews via online tools. Notably, for coherence and consistency, we integrated the specific data collection technology, WeChat video calls, within the Participants section, ensuring a seamless connection with the sample details. |
| S12 | Units of study | We presented the fundamental characteristics of the participants in Table 1. |
| S13 | Data processing | We consolidated these three elements into the Data Analysis section, outlining the procedures for data processing, analysis, and verification. |
| S14 | Data analysis |  |
| S15 | Techniques to enhance trustworthiness |  |
| Results/findings | | |
| S16 | Synthesis and interpretation | We presented the four themes and a conceptual framework derived from them, rooted in the theoretical constructs of Kanter’s theory. Our emphasis on interpretation is consistent with the methodological approach we adopted. |
| S17 | Links to empirical data | We incorporated direct quotes from the interviewees to substantiate pertinent interpretations and arguments, alongside our observational notes, throughout the Findings section. |
| Discussion | | |
| S18 | Integration into prior work, implications, transferability, and contribution(s) to the field | We synthesized our primary findings and juxtaposed them with relevant literature, thereby accentuating the contributions of our study to nursing research and delineating implications for fostering equitable empowerment practices. |
| S19 | Limitations | We acknowledged five research limitations to enhance readers’ understanding of the research and provide insights for future studies. |
| Other | | |
| S20 | Conflicts of interest | We disclosed the absence of potential conflicts of interest in the study. |
| S21 | Funding | We included the funding information on the title page. |
